# Day-4 Lille Score Is a Good Prognostic Factor and Early Predictor in Assessing Therapy Response in Patients with Liver Cirrhosis and Severe Alcoholic Hepatitis

**DOI:** 10.3390/jcm10112338

**Published:** 2021-05-27

**Authors:** Camelia Gianina Foncea, Ioan Sporea, Raluca Lupușoru, Tudor Voicu Moga, Felix Bende, Roxana Șirli, Alina Popescu

**Affiliations:** 1 Department of Gastroenterology and Hepatology, “Victor Babeș” University of Medicine and Pharmacy, Piața Eftimie Murgu 2, 300041 Timișoara, Romania; Foncea.camelia@gmail.com (C.G.F.); isporea@umft.ro (I.S.); raluca_lupusoru@yahoo.ro (R.L.); moga.tudor@umft.ro (T.V.M.); bende.felix@umft.ro (F.B.); sirli.roxana@umft.ro (R.Ș.); 2 Center of Advanced Research in Gastroenterology and Hepatology, “Victor Babeș” University of Medicine and Pharmacy, 300041 Timisoara, Romania; 3 Center for Modeling Biological Systems and Data Analysis, Department of Functional Science, University of Medicine and Pharmacy “Victor Babeș”, 300041 Timișoara, Romania

**Keywords:** alcoholic hepatitis, Lille score, corticosteroid therapy, prognostic scores, alcoholic liver cirrhosis

## Abstract

Lille score at Day 7 (LM7) helps to predict the outcome of patients with severe alcoholic hepatitis (sAH) undergoing corticotherapy. Several scores such as Maddrey’s discriminant function (MDF), MELD, ABIC, and GAHS are used for a 28-day mortality prognosis. Our study aimed to evaluate if the assessment of the Lille score at 4 days (LM4) is as useful as the Lille score at Day 7 (LM7) to predict response to corticosteroids and 28-day mortality and evaluate the utility of severity scores at admission for predicting the prognosis of patients with liver cirrhosis (LC) and severe alcoholic hepatitis (sAH). A retrospective study was performed, and all consecutive patients with AH and MDF > 32 without contraindications to corticosteroids were included. Prognostic scores were evaluated at admission, and 28-day mortality was assessed. Response to corticotherapy was assessed by LM4 and LM7. Results: A total of 55/103 patients with sAH (51.5%) had MDF > 32 and received corticosteroids. There was no difference between the proportion of patients with a responder LM4 versus LM7 (27% vs. 36%, *p* = 0.31). The mean value for LM4 was 0.64 ± 0.3 versus 0.60 ± 0.3 for LM7 (*p* = 0.48). Precisely 90.3% of patients were correctly identified as responders or not by LM4 compared with LM7. The best model for predicting 28-day mortality was composed of MELD and LM4/LM7, with an accuracy of 0.90 for both combinations. Conclusion: LM4 could be used instead of LM7 for predicting response to corticosteroid therapy in patients with sAH and LC, as well as 28-day mortality. Using LM4, we could avoid prolonged use of this therapy and its complications.

## 1. Introduction

Alcoholic hepatitis (AH) is a clinical syndrome that appears due to abusive chronic alcohol consumption. Almost 20% of heavy drinkers will develop AH [1]. It is characterized by the recent onset of jaundice and systemic inflammatory response syndrome (SIRS) and may progress to acute liver failure (ALF) or acute-on-chronic liver failure (ACLF) in patients with underlying liver disease such as cirrhosis [2].

The diagnosis of AH is most often based on clinical grounds. The most important sign is the recent onset of jaundice, within 60 days of the last alcohol intake, in patients known with heavy alcohol use for more than 6 months [3]. The threshold of heavy alcohol use, which could lead to liver injury, is at least three drinks (40 g) daily for women or at least four drinks (60 g) daily for men for more than 5 years [2]. Laboratory characteristics for the diagnosis of AH are serum bilirubin of at least 3 mg/dL, aspartate aminotransferase (AST) between 50 and 400 IU/mL, and an AST/ alanine aminotransferase (ALT) ratio of at least 1.5 [2]. The gold standard in the diagnosis of AH is liver biopsy. However, due to limitations such as coagulation disorders, the presence of ascites, and the unavailability of transjugular biopsy in many centers, based on experts’ opinion, a liver biopsy is recommended only when the diagnosis is uncertain [4,5].

Prognostic scores have been developed in AH to assess the early risk of death in order to establish the severity, need for treatment, and follow-up. The most commonly used scoring systems are Maddrey’s discriminant function (MDF), the model for end-stage liver disease (MELD) score, the age, serum bilirubin, international normalized ratio, creatinine (ABIC) score, the Glasgow alcoholic hepatitis (GAHS) score, and the LILLE score (age, renal insufficiency, albumin, prothrombin time, bilirubin baseline and at 7 days) [6].

MDF is the standard score used to predict AH severity and the need for treatment, using the formula: 4.6 × (Patients PT—Control PT) + TBili [7]. A cut-off value >32 predicts the 28-day mortality of 20–30% and identifies patients with severe alcoholic hepatitis (sAH) in need of treatment [7]. A score <32 predicts a favorable short-time prognosis.

Severe alcoholic hepatitis can lead to life-threatening liver failure, with high mortality (over 50%) and morbidity [8] and a high risk of infections [9]. Furthermore, sAH is becoming more frequent among young people, requires a prolonged hospitalization time, and is one of the most costly diseases; therefore, early recognition and specific treatment are decisive in AH.

Corticosteroid treatment is the most studied in patients with sAH. The analysis of individual data from five randomized controlled trials [10,11,12,13,14], which included 418 randomized patients, confirmed the efficacy of corticosteroids in improving short-time survival in sAH [14]. Patients treated with prednisolone have an early improvement in liver function and an increased short-time survival at 28 days [8]. Response to therapy may be evaluated by the Lille model (LM), a useful tool in the assessment of liver function improvement of patients undergoing corticotherapy [15]. LM is calculated on Day 7 (LM7) of corticotherapy and combines six variables (age, renal insufficiency, albumin, prothrombin time, bilirubin, and evolution of bilirubin at Day 7). According to the cut-off value of 0.45, patients are classified as responders to therapy LM < 0.45 and nonresponders LM ≥ 0.45. In patients with LM7 ≥ 0.45 (nonresponders, almost 40%), the 6-month survival is 25%, and the interruption of corticosteroids is required [15]. To observe a 7-day response to corticosteroids, patients may be hospitalized, which leads to an increased duration of hospitalization and higher healthcare costs.

Systematic screening for infections must be made at admission, as in AH, SIRS criteria may be present even in the absence of a bacterial, viral, or fungal infection. sAH is associated with a high risk of infection, which is one of the major causes of mortality in this setting [9,16]. In the Steroids or Pentoxifylline for Alcoholic Hepatitis (STOPAH) trial, patients with prednisolone had a higher risk of infection (13% vs. 7% in patients without prednisolone), but corticotherapy improved 28-day survival [17]. Louvet et al. demonstrated that infections are not an independent prognostic factor, and early response to therapy seems to be more important for predicting both survival and infection [9].

For patients not responding to corticotherapy, the urge to find another therapy strategy is a priority. Novel therapies and even liver transplantation (LT) are taken into consideration [2]. Therefore, establishing as early as possible if there is a response to corticosteroid treatment is considered a major goal in patients with sAH to avoid unnecessary treatment accompanied by the risk of complications and prolonged hospitalization.

This study aims to evaluate if the assessment of a Lille score earlier, at 4 days (LM4), is as useful as LM7 to predict response to corticosteroids and 28-day mortality and also to evaluate the utility of severity scores at admission for predicting the prognosis of patients with liver cirrhosis and sAH.

## 2. Materials and Methods

### 2.1. Patients

The study is based on a retrospective analysis of patients already diagnosed with liver cirrhosis at admission, admitted with AH between October 2015 and March 2020 in the tertiary Department of Gastroenterology and Hepatology, “Pius Brinzeu”Emergency County Hospital Timisoara, Romania.

All included patients were previously diagnosed with alcoholic liver cirrhosis by well-established criteria such as standard ultrasound with findings suggestive of liver cirrhosis (hepatomegaly and heterogenous liver parenchyma, splenomegaly, signs of portal hypertension), validated with elastography or biological tests with values suggestive of F4 (cirrhosis) and upper digestive endoscopy with the presence of esophageal or gastric varices or portal hypertensive gastropathy. For the etiological diagnosis, other causes were excluded such as hepatitis B or C, autoimmune cirrhosis, and primary or secondary biliary cholangitis.

The diagnosis criteria for AH and inclusion in the study group were: recent onset of jaundice in a patient with a history of heavy alcohol use (~40 g per day for women and ~60 g per day for men for >6 months), <60 days of abstinence before presentation, bilirubin > 3 mg/dL, and an AST/ALT ratio of >1.5. Histological confirmation was not available.

All patients admitted with AH during the established period were included in the study. From the AH initial group, a subgroup of patients with sAH (MDF > 32) was identified and further analyzed. For the final study, we used the following exclusion criteria and established the studied cohort: (1) contraindications to corticosteroids: uncontrolled acute infections, active gastric ulcers, presence of neoplasia, hepatorenal syndrome; (2) presence of chronic underlying liver disease (active hepatitis B or hepatitis C, suspected autoimmune liver disease or drug-induced liver disease).

### 2.2. Clinical and Biological Data

The following variables were recorded at admission during hospitalization at 4 and 7 days and during follow-up: age, gender, approximate alcohol consumption, and biological data (ALT, AST, bilirubin, creatinine, international normal ratio, prothrombin time, albumin, white blood cells).

Complications present at admission or during hospitalization were also recorded (hepatic encephalopathy, presence of ascites, acute kidney injury, gastrointestinal bleeding (GIB), and infections). The diagnosis of liver cirrhosis was previously established and based on clinical, biological, abdominal ultrasound, elastography, and endoscopy criteria.

Systematic screening of infections was performed at admission. In all patients, the following were performed: urine culture, blood culture, chest X-ray, sputum culture, and ascites cultures, where applicable. Infection follow-up was not performed routinely, only when suggestive clinical symptoms appeared.

All patients included in the sAH corticosteroid subgroup received prednisone 40 mg/day, and the 28-day mortality rate was documented for all patients.

The following prognostic scores were calculated in order to find the best predictor of early mortality in sAH, and literature-based cut-off values were used as indicators for severe disease: ABIC > 9 [(age × 0.1) + (serum bilirubin × 0.08) + (serum creatinine × 0.3) + (INR × 0.8)], GAHS ≥ 9 (sum the points assigned for each of the 5 factors: age, white blood cells, urea, prothrombin time ratio, bilirubin), MELD ≥ 21 ((3.8 × loge bilirubin (mg/dL) + 11.2 loge INR + 9.6 loge creatinine (mg/dL) + 6.4) [6].

Response to therapy was assessed by the Lille model at 4 and 7 days, with the formula provided at www.lillemodel.com (accessed on 27 May 2021):

Lille model score = (exp(−R))/(1 + exp(−R)), where R = 3.19 − (0.10 × age) + (0.147 × baseline albumin) + (0.0165 × change in bilirubin level) − (0.206 × creatinine) − (0.0065 × baseline bilirubin) − (0.0096 × prothrombin time) using total serum bilirubin values at Days 4 and 7, respectively. LM4/7 ≥ 0.45 classified the patients into nonresponders, and treatment with prednisone was discontinued. Patients with a LM4/7 < 0.45 continued treatment for 28 days.

### 2.3. Statistical Analysis

Statistical analysis was performed using MedCalc Version 19.3.1. Descriptive statistics were used for clinical, anthropometric, and demographic data of the patients. Numerical variables with a normal distribution are presented as means ± standard deviation, while variables with a non-normal distribution are presented as median values and range. The Kolmogorov–Smirnov test was used for testing the distribution of numerical variables. Qualitative variables were presented as numbers and percentages. Parametric tests (*t*-test) were used for the assessment of differences between numerical variables with a normal distribution, and nonparametric tests (Mann–Whitney or Kruskal–Wallis tests) for variables with a non-normal distribution. The Chi-square (χ^2^) test (with Yates’ correction for continuity) was used for comparing proportions expressed as percentages. Furthermore, 95% confidence intervals were calculated for each predictive test, and *p* < 0.05 was considered significant for each statistical test. The Pearson test was used for correlations. Areas under the receiver-operating characteristic (AUROC) curves were calculated for the mortality-predicting scores, and *z*-scores were calculated when comparing ROC curves.

## 3. Results

### 3.1. Baseline Characteristics

**Figure 1:**
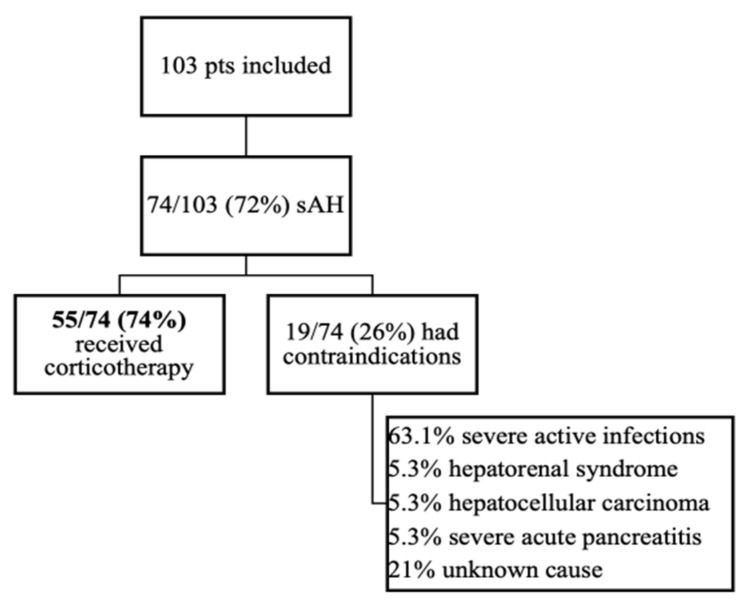
Patients included in the study.

A total of 103 patients were diagnosed with AH between October 2015 and March 2020 in the tertiary Department of Gastroenterology and Hepatology, out of which 74 (72%) had MDF > 32 and were classified as sAH and thus included in the final analysis. From 74 patients with MDF > 32, only 55/74 (74%) received corticosteroids, while 19/74 (26%) had contraindication (Figure 1) and received supportive therapy, consisting of intravenous fluid electrolyte, mineral, and vitamin supplementation, including the replacement of potassium, multivitamins, and thiamine.

All included patients had liver cirrhosis: 55/74 (74%) were Child–Pugh Class C. Because the study was retrospective, we could observe that patients with sAH superimposed on acute pancreatitis, liver cirrhosis with hepatocellular carcinoma, or the development of hepatorenal syndrome were not given corticotherapy. In the subgroup of patients with contraindications, 12/19 (63.1%) presented infections at presentation. Antibiotherapy was initiated, but the control of infections was not obtained, so corticotherapy was not initiated.

In the studied subgroup that received corticosteroids, 18/55 (32%) presented minor infections at presentation, corticosteroids were initiated immediately after antibiotherapy or together with antibiotics, and 4/55 (7.3%) presented infections after corticotherapy was initiated.

Baseline characteristics are shown in Table 1. Regarding the severity scores, 18 patients out of 55 receiving prednisone had ABIC > 9, 31 patients had GAHS ≥ 9, and 48 patients had MELD ≥ 21 (Table 1).

### 3.2. Comparison between LM4 and LM7

In the sAH corticosteroid subgroup, the mean value for the Lille score at 4 days was 0.64 ± 0.3 vs. 0.60 ± 0.3 for the Lille score at 7 days (*p* = 0.48). There was no difference in our study group between the proportion of patients with a responder Lille score value at 4 days versus 7 days (27% vs. 36%, *p* = 0.31). 15/55 (27%) patients were responders at LM4, and 20/55 (36%) were responders at LM7. From the nonresponders at LM4, 5/55 (9%) became responders at LM7.

The correlation between LM4 and LM7 was r = 0.94, *p* < 0.0001, and R^2^ = 0.88, meaning that 88% of the values of LM4 were in concordance with the values of LM7.

**Figure 2:**
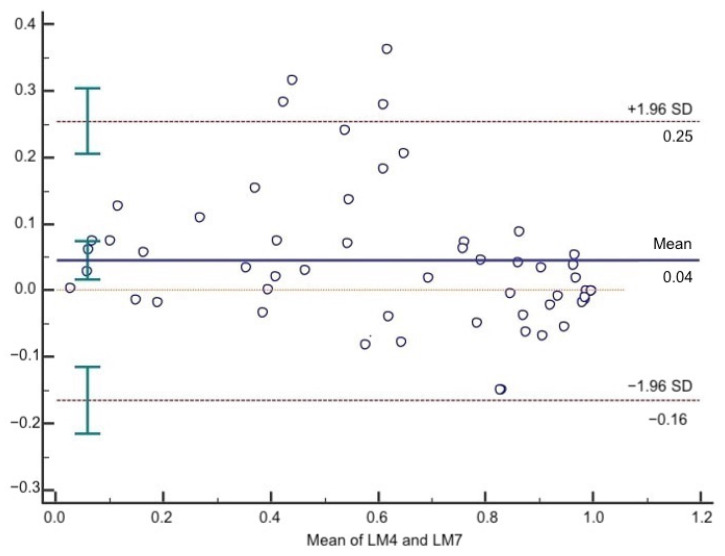
Bland–Altman plot of LM4 and LM7. The solid line represents the mean of the difference. The dashed lines define the LOA. The 95% upper and lower LOA were 0.25 and −0.16, respectively.

According to the Bland–Altman test, the mean difference between LM4 and LM7 was 0.04. The 95% upper and lower LOA were 0.25 and −0.16, respectively (Figure 2).

### 3.3. Performance of LM4 and LM7 in Predicting 28-Days Mortality

**Figure 3:**
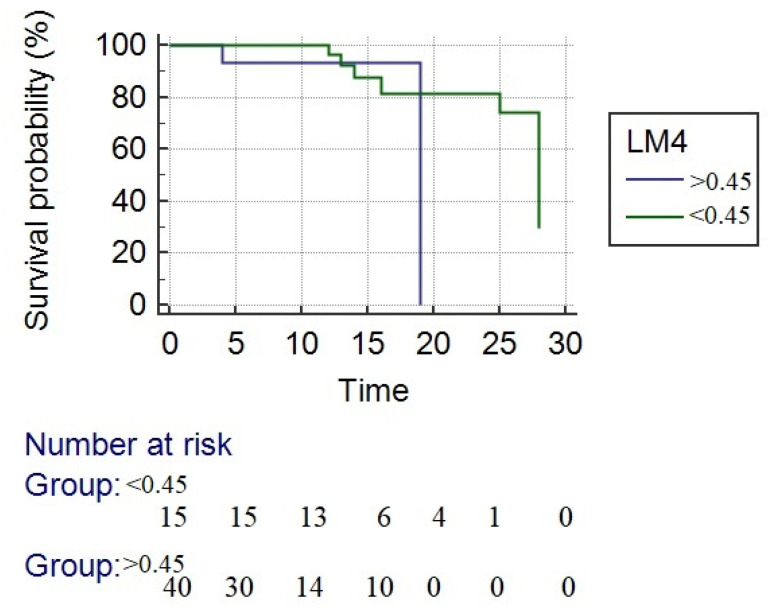
Kaplan–Meier survival analysis. The blue line represents the nonresponders, and the green line represents the responders to corticotherapy. The survival rate at 28 days in responders according to LM4 criteria was significantly higher compared with nonresponders (mean survival time—24.3 days vs. 18 days (*p* = 0.04)).

By using LM4 and LM7 with a cut-off value > 0.45 to separate responders from nonresponders, the 28-day mortality was higher in nonresponders (LM4 13.3% and LM7 31%, *p* = 0.22) than in responders (LM4 30% and LM7 15%, *p* = 0.29), respectively. Precisely 90.3% of patients were correctly identified as responders or not by LM4 compared with LM7. The survival rate at 28 days in responders according to LM4 criteria was significantly higher compared with nonresponders (86.7% vs. 70%, *p* < 0.0001). The mean overall survival was 24.3 days. For responders, the mean survival was 25.1 days and for nonresponders 18 days, responders with a significantly longer survival time (*p* = 0.04) (Figure 3).

**Figure 4:**
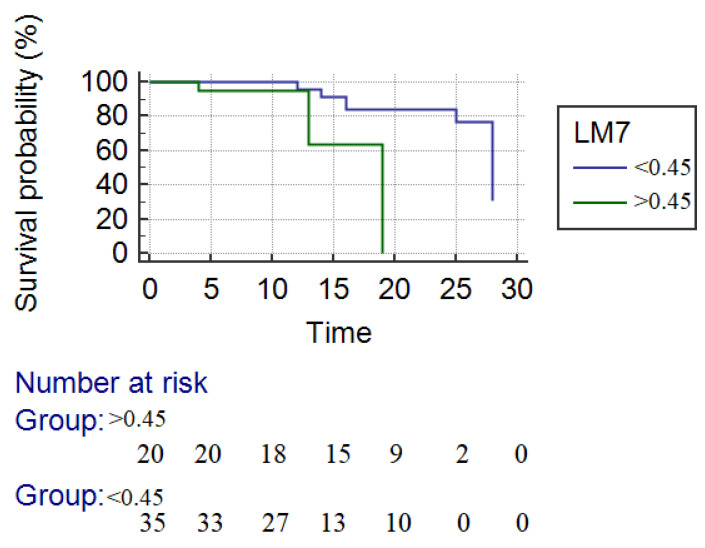
Kaplan–Meier survival analysis. The blue line represents the responders, and the green line represents the nonresponders to corticotherapy. The survival rate at 28 days in responders according to LM4 criteria was significantly higher compared with nonresponders (mean survival time—25.5 days vs. 16.3 days (*p* = 0.04)).

The mean overall survival was 24.3 days. For responders, the mean survival was 25.1 days and for nonresponders 16.3 days, responders with a significantly longer survival time (*p* = 0.01) (Figure 4). The survival rate at 28 days in responders according to LM4 criteria was significantly higher compared with nonresponders (85.0% vs. 61%, *p* = 0.04).

### 3.4. Predictors for Mortality

In univariate analysis, the MELD score, the MADDREY score, and LM4 were associated with 28-day mortality. A Lille score at Day 4 greater than 0.45 can raise the risk of mortality by 7.2 times (Table 2).

### 3.5. Comparison of Different Predicting Scores for Mortality

**Figure 5:**
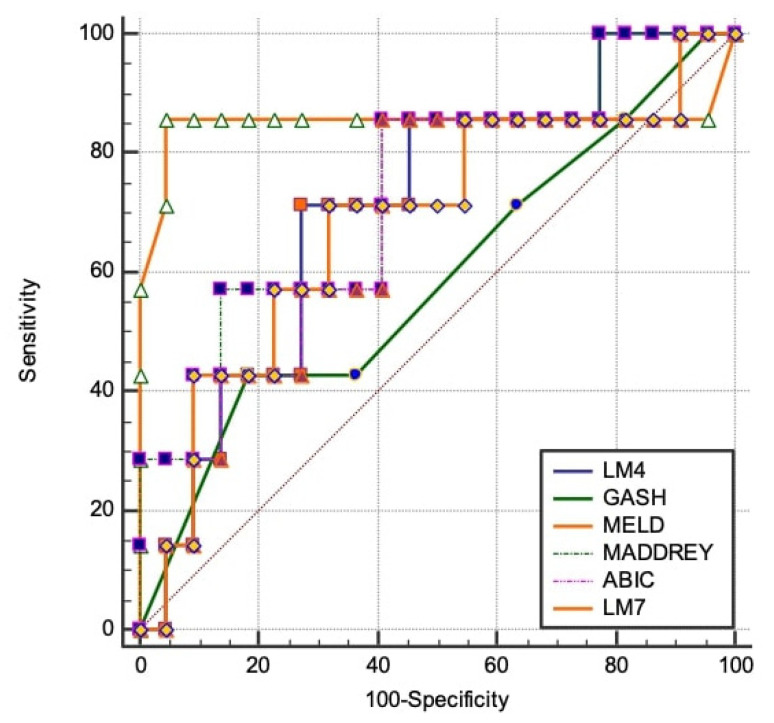
Comparison of AUROCs in order to find the best model for predicting 28-day mortality. The best performance to predict mortality was observed in the MELD score with an AUC of 0.85, followed by the Maddrey score with an AUC of 0.74 and by LM7 and LM4 with AUCs of 0.68 and 0.67, respectively.

By using the literature-based cut-off values as indicators for severe disease (ABIC > 9, GAHS ≥ 9, MELD ≥ 21, LM4/LM7 ≥ 0.45, and MDF>32), the best performance to predict mortality (Table 3) was observed regarding the MELD score with an AUC of 0.85, followed by the Maddrey score with an AUC of 0.74 and by LM7 and LM4 with AUCs of 0.68 and 0.67, respectively. Lower performance was observed for the ABIC and GAHS scores, with AUCs of 0.64 (*p* < 0.0001). There was no difference between the performance of LM4 vs. MELD, MADDREY, and LM7 (*z*-score = 0.58, *p* = 0.64) (Figure 5).

When combining scores to find the best model for predicting 28-day mortality, the best model included the MELD score, LM4, and LM7 with an accuracy of 0.90 for both combinations. MELD + MADDREY had an accuracy of 0.86, while MELD + MADDREY + LM4/7 had an accuracy of 0.87.

## 4. Discussion

Current treatment strategies are limited in sAH. Corticosteroids remain the only therapeutic option and the most widely used treatment, resulting in decreased mortality at one month by 14% [18]. The largest, multicentric, double-blinded, randomized trial performed in this topic, Steroids or Pentoxifylline for AH (STOPAH) [17], evaluated the benefit of treatment with corticosteroids vs. pentoxifylline in sAH. A total of 1103 patients from 65 centers were included, and the results showed that pentoxifylline did not improve survival in patients with sAH, while corticosteroids significantly improved survival after 28 days compared with placebo but with no benefits for the 90-day survival rate. Current guidelines [2] recommend the use of corticosteroid treatment for sAH in the absence of contraindications, such as active and severe infections, impaired renal function, and gastrointestinal bleeding.

In our cohort, 18/55 (32%) of patients presented minor infections at presentations, and corticotherapy was possible after effective antibiotherapy. Louvet et al. showed that infections are frequent in patients with AH, but treatment can be continued, and the most important factor in the short-time survival of these patients is the response to therapy. The mentioned study demonstrated that the probability of being infected after corticosteroids is lower in responders than in nonresponders (11.1% vs. 42.5%), with a median time for infection development of 14 days [9]. Regarding these findings, we can suggest that limitation of corticosteroids exposure could improve outcomes in nonresponders by reducing immunosuppression, especially in a cohort with a high risk of infection such as patients with liver cirrhosis.

In sAH treatment, early change in bilirubin is essential, and studies have shown that a lack of improvement of bilirubin after 7 days of treatment shows futility [19]. Therefore, it is important to stratify patients at risk of mortality at baseline and after initiation of therapy. At baseline, MDF was mostly used, but MELD shows better performance in predicting mortality [20]. In our univariate analysis, the MELD score, MDF, and LM4 were associated with the 28-day mortality. There were no differences between the performance of LM4 and MELD, MADDREY, and LM7 (*z*-score = 0.58, *p* = 0.64). A Lille score at Day 4 greater than 0.45 can raise the risk of mortality by 7.2 times.

Patients with liver cirrhosis have a higher risk of infections. Alcoholic hepatitis superimposed and treatment with corticosteroids increases the risk of infections and, therefore, the risk of mortality. Early identification of response to corticosteroid treatment is thorough, and finding the best predictor model for mortality in these patients is important in order to avoid unnecessary treatment, which could worsen the prognosis. The MELD and MDF scores are static, evaluating patients at baseline, while the Lille score is dynamic, following changes after one week of treatment [21]. Thus, the idea of the utility of scores could be higher by combining them. Louvet et al. [22] combined static scores such as Maddrey, MELD, and ABIC with the dynamic score Lille and demonstrated that the MELD + Lille score can better predict outcomes of patients with AH compared with either model alone. In terms of the study we are referring to, our data are very similar regarding the survival, 75%, and furthermore, as shown in our analysis, the best model for predicting mortality at 28 days was the one that included the MELD score and LM4 or LM7, with an accuracy for both of 0.90. MELD + MDF had an accuracy of 0.86, while MELD + MDF + LM4/7 had an accuracy of 0.87.

A recent multicenter study [23], including 163 patients receiving corticotherapy, had similar findings. In addition to our study, predictors for 90-day mortality were assessed. By calculating LM at Day 4 with the same calculator, using Day 4 bilirubin instead of Day 7, they demonstrated a 91.1% agreement between LM4 and LM7 to predict response (κ = 0.82, *p* < 0.001) for corticosteroids, and LM4 was an independent predictor of mortality at 28 and 90 days in multivariate analysis. In the mentioned study, MDF and ABIC had the best performance in predicting 28-day mortality with an AUC of 0.80 and no differences between LM4 and LM7, while in our study, the best performance to predict mortality was observed regarding the MELD score, with an AUC of 0.85, followed by the Maddrey score, with an AUC of 0.74, and LM7 and LM4, with AUCs of 0.68 and 0.67, respectively. Furthermore, we demonstrated that the correlation between LM4 and LM7 was r = 0.94, *p* < 0.0001, and R^2^ = 0.88, meaning that 88% of the values of LM4 were in concordance with the values of LM7, and we combined scores to find the best model for predicting 28-day mortality in a cohort of patients with alcoholic hepatitis and liver cirrhosis.

From the nonresponders by LM4, 5/55 (9%) became responders by LM7. By analyzing the subgroup of nonresponders by LM4 who became responders at 7 days, we found out that the common element was the presence of infections (four in five patients) for which antibiotherapy was administered together with corticotherapy.

In a burden of illness study performed by Thompson et al. in the United States [24], the authors demonstrated that AH is a highly expensive disease, given the need for multiple and prolonged hospitalizations. In our study, the mean hospitalization duration was 13.65 ± 7.5 days.

The present study has some limitations, such as the small number of patients and the lack of liver biopsy for a definite diagnosis of AH. However, in our study, strict clinical criteria were used for the positive diagnosis of AH, and it must be considered that liver biopsy is indicated in diagnostic uncertainty [5,25]. A small cohort of patients was finally included, and in our retrospective analysis, all patients with alcoholic hepatitis also presented alcoholic liver cirrhosis at admission, a special group of patients who need more attention due to complications that may occur.

By assessing LM at 4 days, not only would responder patients benefit from early discharge from the hospital, but the greater benefit could be for the nonresponders. Early identification of failure to corticosteroids could allow us to resume investigations in order to find a reason for lack of response. The poor outcome in these patients raises the issue of early assessment for liver transplantation (LT) [26]. Evident improvement in survival of patients undergoing early LT was observed [27].

In conclusion, our study showed that LM4 is as accurate as LM7 in predicting response to corticosteroids and 28-day mortality in patients with severe AH. Furthermore, a combination of MELD plus LM4 or LM7 better predicts mortality at 28 days. These findings could decrease the risk of corticotherapy-related complications, spare health care costs for sAH-hospitalized patients, and most promisingly, save 3 days in the evaluation of highly selected patients for LT. These data are promising and need to be validated in a larger cohort of patients.

## Figures and Tables

**Table 1:** Patient characteristics.

Characteristics	Total Patients (N = 74)	Corticosteroid Subgroup (N = 55)	No Corticosteroid Subgroup (N = 19)	*p*-Value
Age (years)	53.5 ± 9.15	53.0 ± 8.8	54.8 ± 10.2	0.50
Gender (male)	61/74 (82.4%)	46/55 (84%)	15/19 (79%)	0.65
Hospitalization days	12.8 ± 7.8	11.0 ± 8.0	10.0 ± 6.4	0.17
Death rate	22/74 (29.7%)	14/55 (25%)	8/19 (42%)	0.70
**Biological parameters**				
WBC (×10^9^/L)	11.8 ± 5.4	10.6 ± 5.6	9.4 ± 4.6	0.10
Platelets (×10^9^/L)	79.0 ± 68.0	84.5 ± 70.0	87.0 ± 69.0	0.64
INR	2.0 ± 0.7	1.8 ± 0.8	2.1 ± 0.4	0.53
PT (s)	19.0 ± 10.6	20.5 ± 6.6	-	0.34
Creatinine (mg/dL)	1.1 ± 0.7	1.1 ± 0.8	0.9 ± 0.6	0.59
Bilirubin (mg/dL)	14.0 ± 8.0	16.0 ± 8.0	13.2 ± 7.7	0.16
Albumin (mg/dL)	2.1 ± 0.5	2.0 ± 0.4	2.0 ± 0.7	0.98
**Severity scores (mean values)**				
MDF	63.1 ± 29.3	69.0 ± 33.3	60.0 ± 25	0.28
Child–Pugh C	55/74 (74.3%)	41/55 (75%)	14/19 (74%)	0.99
MELD	25 ± 6.5	26.0 ± 6.0	24.6 ± 4.9	0.36
ABIC	8.4 ± 1.5	8.5 ± 1.6	8.9 ± 1.4	0.33
GAHS	8.37 ± 1.3	9.0 ± 1.5	9.0 ± 1.2	0.99
**Complications at presentation**				
Acute renal failure (Cr >1.5 mg/dL)	11/74 (15%)	7/55 (13%)	4/15 (27%)	0.22
Gastrointestinal bleeding	3/74 (4%)	3/55 (5%)	-	0.78
Infections	37/74 (50%)	25/55 (45%)	12/19 (63%)	0.48

Numerical variables with a normal distribution are presented as the mean value ± standard deviation, while variables with a non-normal distribution are presented as median values and range intervals. N—number; WBC—white blood cells; INR—international normalized ratio; PT—prothrombin time; MDF—Maddrey’s discriminant function; MELD—model for end-stage liver disease; ABIC = age, serum bilirubin, international normalized ratio, creatinine score; GAHS = Glasgow alcoholic hepatitis score.

**Table 2:** Univariate logistic regression of factors associated with mortality.

Parameter	OR (95% CI)	*p*-Value
MELD	1.27 (1.05 to 1.54)	0.01
MADDREY	1.01 (1.0 to 1.03)	0.04
LM4	7.26 (0.62 to 84.02)	0.01
LM7	7.62 (0.86 to 67.32)	0.06
ABIC	1.30 (0.87 to 1.92)	0.18
GAHS	1.47 (0.93 to 2.33)	0.09
Age	1.01 (0.94 to 1.08)	0.66
Male gender	1.51 (0.15 to 1.90)	0.03
AST	1.00 (0.99 to 1.00)	0.51
ALT	0.99 (0.99 to 1.00)	0.89
TB	1.04 (0.94 to 1.14)	0.42
Albumin	0.18 (0.03 to 1.09)	0.06

LM7 = Lille score at 7 days; LM4 = Lille score at 4 days; ABIC = age, serum bilirubin, international normalized ratio, creatinine score; GAHS = Glasgow alcoholic hepatitis score; MELD = model for end-stage liver disease; AST = aspartate aminotransferase; ALT = alanine aminotransferase; TB = total serum bilirubin; OR = odds ratio; CI = confidence interval.

**Table 3:** Performance of different scores in predicting mortality at 28 days.

Scores	AUC	SE	95% CI
MADDREY	0.74	0.11	0.54 to 0.88
MELD	0.85	0.13	0.67 to 0.95
LM4	0.67	0.12	0.47 to 0.83
LM7	0.68	0.13	0.48 to 0.84
ABIC	0.64	0.08	0.50 to 0.76
GAHS	0.64	0.08	0.49 to 0.76

AUC = area under the receiver-operating curve; LM4 = Lille score at Day 4; LM7 = Lille score at Day 7; SE = standard error; CI = confidence interval; MELD = model for end-stage liver disease; ABIC = age, serum bilirubin, international normalized ratio, creatinine score; GAHS = Glasgow alcoholic hepatitis score.

## Data Availability

The data underlying the findings of the study are available on request to the corresponding author (e-mail address: alinamircea.popescu@gmail.com).
